# Family Impacts on Self-Esteem in Chinese College Freshmen

**DOI:** 10.3389/fpsyt.2017.00279

**Published:** 2017-12-12

**Authors:** Jingyu Shi, Lu Wang, Yuhong Yao, Na Su, Xudong Zhao, Fazhan Chen

**Affiliations:** ^1^East Hospital, Tongji University School of Medicine, Shanghai, China; ^2^Students Counseling Center, Tongji University, Shanghai, China; ^3^Tongji University School of Medicine, Shanghai, China

**Keywords:** self-esteem, family function, only child, grandparenting, college freshmen

## Abstract

This study examined the impact of family function and family-related factors, such as being an only child, grandparenting, income, and family relationship on the self-esteem in college students who are in the transitional period from late adolescence to emerging adulthood. The participants were 2001 Chinese college freshmen with the age from 16 to 20 years. Data were collected by using the family assessment device (FAD), the Rosenberg Self-esteem Scale, and self-report of family information. Comparison analysis indicated that the students from one child families, harmonious families, from families with higher income, or raised by their parents without the experience of grandparenting are more likely to show high self-esteem than their counterparts. Moreover, a multiple regression showed that dimensions of FAD such as role, communication, behavioral control, and problem solving predicted level of self-esteem of the college students, ranging from 13.2 to 17.9% variance. The results of this study showed that the self-esteem of the college freshmen is highly correlated with their family’s performance. Therefore, the program focusing on improving family functioning is needed, in order to enhance the self-esteem of the young people and hence contribute to promoting the mental health of them.

## Introduction

The college years are a developmentally crucial period. The college students are making the transition from late adolescence to emerging adulthood (1). During this period, the young people are faced with many challenges and have a wide range of needs. On one side, the youth will inevitably undergo lots of changes in their transition from high school students to college students. They need to cope with changes independently and manage their college life to adapt to the new environment (2). On another side, they are confronted with the accomplishment of psychosocial developmental tasks in adolescence and coping with new psychosocial developmental challenges in early adulthood (3). Therefore, college students are prone to inner conflicts and troubles. Mental health is one of the most important aspects for college students to be competent for college life (4). The previous studies show that adaptation problems among college freshmen were common (5, 6); There were one third of college freshmen showing mental health problems, which could be associated with long-term adverse outcomes in later adulthood (4, 7).

Self-esteem describes the positive appraisal and experience concerning self-value, which an individual obtains in the socialization process (8). Self-esteem is an important trait of the self system, and it has a positive influence on the individual’s mental health and personality development (8). According to Maslow (9), people with a sense of high self-esteem are more confident, competent, and therefore more successful, while those with sense of low self-esteem tend to feel inferior, frustrated, hopeless, and even get mental illness. The findings of many studies also show that people with high self-esteem have high level of mental health and self consistency (10, 11). It has been found that low level of self-esteem is highly correlated with depression (12, 13). Liu et al. (14) reported that self-esteem regulated the interaction between human and environment and played a role of “buffering anxiety.” In addition, self-esteem may contribute to the positive coping and the realization of goals, whereas low self-esteem may cause avoidance (15, 16). Self-esteem is one of the most important protective factors for the mental health of college students (17, 18).

The development of self-esteem is related to the living environment and cultural background of individuals. Family is the most direct and important environment for individual growth and socialization; the significant developmental tasks of individuals are resolved within the family (19). College students are in the transitional stage from late adolescence to emerging adulthood; although they depart from their families, they still have an emotional tie to their family, and the influence of the family on the individuals has not weakened. The parents–children relationship models constantly play a role on the psychosocial development and adaptation of the college students (20). And moreover, while the youth leave home, the family members should adjust their models of relationship. On one side, the college students need autonomy and independence to separate with their parents; on the other side, they still need the close emotional bonding with their parents to feel safety when they explore their inner and external world (21). Several studies have shown that there was a significant correlation between the development of adolescents’ self-esteem and parental bonding style, and the researchers have found that insufficient care and excessive control by parents lead to depressive symptoms and low self-esteem (22–24). Other findings suggest that high self-esteem and self achievement of the adolescents positively associated with high levels of intimacy and emotional expression in the family (25); the closeness of family members and the expectations of parents will influence the college freshmen’s self-esteem and self-concept (26). There is also evidence that poor family functioning is likely to negatively influence adolescents’ physical and emotional development, potentially leading to feelings of being neglected, insecurity, and uncertainty, which in turn, impair offspring’s self-esteem. In view of reported associations between family functioning and self-esteem (27–30) and increasing evidence for poor self-esteem as a predictor to mental disorders in youth (12, 13), family functioning features can be considered as an important factor in promoting lower self-esteem in youth. Moreover, both family functioning and self-esteem could be promising targets to protect offspring’s mental health.

Although the literature has shown that family environment, family functioning, and parental patterns could influence the self-esteem of the offspring, there is still a large unknown field on this research topic. First, results are often based on small or clinical samples and focused on children and adolescents, restricting the use of more complete statistical examinations and generalization to the general population, especially to the college students who are in the transitional stage into emerging adulthood. Second, Chinese society has some unique familial features which may have an impact on the self-esteem of college students. One remarkable feature is the so-called “grandparenting.” This makes a significant Chinese characteristic for Chinese families as the grandparents often take care of the grandchildren from birth to kindergarten or even up to primary school. As a result, children might establish a closer bonding to their grandparents than to their parents during early childhood. In the following, we will show what potential impacts grandparenting has on the self-esteem of individuals. In this study, we considered grandparenting as the experience of leaving from the parents and being raised by grandparents for at least 1 year in the childhood. Considering the fact that in China quite a lot of college students are only child, the following study additionally will discuss the different levels of only child college students’ self-esteem in comparison with students who have siblings. Therefore, exploring these findings with an Asian cultural perspective can support the external validity with meaningful evidence.

In summary, the aim of the current study is to investigate the relationship between different aspects of family functioning and self-esteem in Chinese college freshmen. We also go beyond previous studies in examining impacts of the unique factors of Chinese families (like only child, grandparenting,…) on the self-esteem of college freshmen. It is hoped that the findings of this study could be a short step toward promoting the mental health of college students.

## Materials and Methods

### Participants

A sample of 2,001 freshman students (1,082 males, 919 females) was recruited during the academic year 2014–2015 at a university in Shanghai, which is a large city in the eastern part of China. The age range was between 16 and 20 years (mean age = 18.51 years, SD = 0.81 years). Behavioral protocol was approved by the Institutional Review Board of Tongji University. Written informed consent was obtained from all the participants prior to the study.

### Measures

#### Family Assessment Device (FAD)

The FAD, constructed according to the McMaster model of Family Functioning (MMFF), is used to assess the structural, transactional, and organizational characteristics of a family. It includes six subscales, assessing six dimensions of MMFF; they are problem solving, communication, roles, affective responsiveness, affective involvement, and behavior control. Problem solving refers to the capability of the family to resolve problems in order to keep the functional effectiveness of the family; communication is defined as the way of exchanging information among family members; roles reflects whether the family assigns clear and equitable tasks to family member to guarantee implementation of family functions such as providing resources and support; affective responsiveness assesses to which extent the family members are able to emotionally react to stimulation; affective involvement reflects to which extent the family members show interest and concern to each other; and behavior control assesses the behavioral models that the family establishes in different situations. The FAD scores each of these subscales and also a general family functioning scale. The scale consists of 60 statements about a family and requires respondents to evaluate how well each statement expresses their own family status. Each item was scored at the 4-point Likert scale, with the option to answer “1 = very like my family” to “4 = totally unlike my family.” Sample items for example included, “If someone is in trouble, the others become too involved,” “Making decisions is a problem in our family.” In these analyses, higher scores on the FAD scales reflect dysfunctional family functioning. Shek (31, 32) has tested the validity and reliability of the scale in Chinese population. The coefficient alpha ranged from 0.53 to 0.94.

#### Rosenberg Self-Esteem Scale (RSES)

Self-esteem was evaluated using the RSES (33). It is a self-rating scale consisting of 10 items, using 4-point Likert scale to rate, with option from “1 = strongly disagree” to “4 = strongly agree.” Sample items are such as “I can do things as well as most people” and “I take a positive attitude toward myself.” The total score ranges from 10 points (low level of self-esteem) to 40 points (high level of self-esteem). The scale was shown to have good reliability and validity (34, 35). In the current study, the Cronbach alpha coefficient of RSES was 0.84.

#### Self-Report of Family Information

Family information was collected, and the information included: (1) “whether only child” with the binary answer “0” for “No” and “1” for “Yes”; (2) “family relationship” assessed with multiple categorical answer “1 = harmonious,” “2 = not bad,” “3 = conflicting,” “4 = parents divorced.” The construction of the classifications was based on the results of qualitative interview of students; (3) “whether raised by grandparents” with the binary answer “0” for “No” and “1” for “Yes”; and (4) “family economic status” assessed by monthly family income with the classification from “1” for “<2,000 RMB” (ca. 325 US$) to “4” for “>10,000 RMB” (ca. 1,627 US$). The responders were asked to choose one answer from the multiple choices. All the questionnaires employed in this study were in Chinese language.

### Procedure

The participants were instructed to finish up a questionnaire survey including measures of family functions, self-esteem and family information in a quiet classroom after signing informing consent. It took approximately 15 min for them to complete all the instruments.

### Data Analysis

All statistical procedures were conducted using the Statistical Package for the Social Sciences (SPSS 17.0). One-way ANOVA and independent sample *t*-test were employed to compare the level of self-esteem among the different groups divided according to the self-reported data referring to family information; Pearson correlation and multivariate stepwise regression were conducted to explore how levels of self-esteem and family functions as well as other family factors related.

## Results

### Comparison of the Level of Self-Esteem among Groups with Different Family Variables

Between the male and female students there was no statistical difference on the level of self-esteem (*P* > 0.05). The students who were only child scored higher on self-esteem than students with siblings (*P* < 0.001). Students reporting different quality of family relationship were rated on a different level of self-esteem (*P* < 0.001), bivariate comparisons showed that the students from harmonious families were rated higher on self-esteem than the students from the conflicting families (mean difference = 2.465, *P* < 0.05) and from the families, in which the family relationship was “not bad” (mean difference = 1.077, *P* < 0.05); the students with experience of grandparenting scored lower on self-esteem than students raised by parents (*P* = 0.001). Students from families with higher income scored higher on self-esteem than students from families with lower income (*P* < 0.001), bivariate comparisons showed that the students reporting a monthly family income of more than 10,000 RMB (ca. 1,627 US$) were rated higher on self-esteem than the students reporting a monthly family income less than 2,000 RMB (ca. 325US$) (mean difference = 1.602, *P* < 0.05), 2000–4999 RMB (325-813US$) (mean difference = 0.740, *P* < 0.05) and 5,000–9,999 RMB (813–1,627 US$) (mean difference = 0.681, *P* < 0.05). Table 1 presents frequency, mean, SD, and statistic value of comparison.

**Table 1:** Comparison of the level of self-esteem among groups with different family variables.

Variables		*n*	Self-esteem	*t*/*F*	*P*
Gender	Female	916	30.30 ± 4.132	0.214	0.830
	Male	1,073	30.34 ± 4.097		

Only child	Yes	1,539	30.50 ± 0.105	3.656	0.000
	No	434	29.68 ± 0.192		

Family relationship	Harmonious	1,659	30.50 ± 4.077	8.213	0.000
	Not bad	200	29.42 ± 4.322		
	Conflicting	31	28.03 ± 4.086		
	Parents divorced	73	29.62 ± 3.755		

Raised by grandparents	No	1,394	30.52 ± 4.031	−3.226	0.001
	Yes	582	29.86 ± 4.267		

Monthly family income	<2,000 RMB	168	29.27 ± 4.459	7.528	0.000
	2,000–4,999 RMB	500	30.13 ± 3.932		
	5,000–9,999 RMB	680	30.19 ± 4.014		
	>10,000 RMB	537	30.87 ± 4.170		

### Correlation between Family Variables and Self-Esteem

The bivariate correlation analysis showed that the level of self-esteem negatively correlated with the score of all the seven subscales of the FAD (*P* < 0.001). The other family variables such as whether only child, family relationship, grandparenting, and family income also lightly correlated with self-esteem (*P* < 0.001). The correlation matrix between level of self-esteem and family variables are presented in Table 2.

**Table 2:** Correlation analysis between family variables and self-esteem.

	PS	CM	RL	AR	AI	BC	GF	GENDER	WSC	FR	GP	MFI
SE	−0.280**	−0.361**	−0.362**	−0.306**	−0.319**	−0.265**	−0.360**	−0.005	0.072**	−0.089**	−0.072**	0.101**
PS		0.563**	0.445**	0.454**	0.355**	0.326**	0.616**	−0.053*	−0.083**	0.189**	0.072**	−0.043
CM			0.592**	0.662**	0.585**	0.285**	0.757**	−0.100**	−0.060**	0.228**	0.064**	−0.071**
RL				0.546**	0.616**	0.420**	0.652**	−0.117**	−0.153**	0.190**	0.080**	−0.212**
AR					0.531**	0.324**	0.674**	−0.203**	−0.112**	0.202**	0.044	−0.117**
AI						0.365**	0.679**	−0.122**	−0.078**	0.178**	0.074**	−0.092**
BC							0.386**	−0.061**	−0.077**	0.118**	0.020	−0.109**
GF								−0.134**	−0.065**	0.303**	0.083**	−0.091**
GENDER									0.059**	−0.018	−0.016	0.209**
WSC										−0.040	−0.075**	0.275**
FR											0.077**	−0.072**
GP												−0.002

*SE, self-esteem; PS, problem solving; CM, communication; RL, role; AR, affective responsiveness; AI, affective involvement; BC, behavioral control; GF, general family functioning; WSC, whether single child; FR, family relationship; GP, grandparenting; MFI, monthly family income*.

*P < 0.05.

**P < 0.01.

### Multivariate Regression Analysis

To explore the family impact factors on level of self-esteem, the multivariate stepwise regression analysis was run for those independent family variables that showed a significant relationship with the level of self-esteem as measured by bivariate correlation. Only data of participants who had provided the complete data were entered into multivariate regression analysis. Four variables were selected into equation on α = 0.05 level. There were role (RL), communication (CM), behavioral control (BC), and problem solving (PS). Regression coefficients were ranging from β = 0.059 to β = 0.187. Family functioning predicted level of self-esteem, ranging from predictions of roles with 13.2% variance (*R*^2^ = 0.132; *F* = 280.004; *P* < 0.001) to predictions of problem solving with 17.9% variance (*R*^2^ = 0.179; *F* = 99.861; *P* < 0.001). The coefficients of regression are presented in Table 3.

**Table 3:** Results of multiple regression analysis between family functioning and self-esteem.

Variable	*R*^2^	*F*	β	*t*
RL	0.132	280.004**	−0.178	−6.357**
CM	0.164	180.086**	−0.187	−6.460**
BC	0.177	131.173**	−0.116	−4.903**
PS	0.179	99.861**	−0.059	−2.248*
AR			−0.032	−1.087
AI			−0.051	−1.759
GF			−0.043	−1.155
WSC			0.011	0.521
FR			0.017	0.770
GP			−0.040	−1.900
MFI			0.042	1.913

*PS, problem solving; CM, communication; RL, role; AR, affective responsiveness; AI, affective involvement; BC, behavioral control; GF, general family functioning; WSC, whether single child; FR, family relationship; GP, grandparenting; MFI, monthly family income*.

*P < 0.05.

**P < 0.01.

## Discussion

The present study was designed to investigate the crucial role of family factors and family functioning in the level of self-esteem in a sample of Chinese college freshman. First, through comparison of the level of self-esteem between different groups according to the family factors, it is found that the only child was rated significantly higher on self-esteem than the students with siblings. The possible reasons can be that the only child gain more attention from their parents, the parents usually have higher expectations on the only child, hence they can get more support and resources from their parents, which could contribute to the higher level of self-esteem (36, 37). The results of comparison analysis also suggested that students raised by their grandparents showed lower levels of self-esteem than students who were raised by their parents. Grandparenting is a common phenomenon in China, and it is related to the Chinese social and cultural background. On the one hand, both of the parents are too busy working to take care of their children; or the parents work in another city far away from their hometown, so the children are sent to the grandparents to be raised until they go to kindergarten or primary school. On another hand, Chinese culture is a collectivistic culture, deeply influenced by Confucianism, while western culture is individualistic. Collectivism stresses common interests, coherence, cooperation, and interdependence. This culture emphasizes the importance of extended families and collectives, where everyone needs to take responsibility for other members of their organization (38). Therefore, in China, many grandparents will take the initiative to take care of grandchildren after retirement. As a result, children become less attached to their parents in their early childhood, and instead become more attached to their grandparents (39, 40). If lack of parental care and companionship during the first years of life, it means that when children return to their parents, the parents and children are estranged from each other, which may affect the children’s self-evaluation and self-awareness (40–44). Researchers (40, 41, 44) found that for young people, the attachment to parents has a significant impact on self-competence and self-liking. College students make up a special group of young people: physically and psychologically they are in the transition stage from adolescence to adult, they have the psychological need to feel as an adult, but on the other hand they still depend on their parents. Wang (40) suggested that in the process toward independence, the college students who did not establish intimacy with their parents felt more sense of insecurity and loneliness. In addition, we also found that the quality of family relationship may influence the level of self-esteem. The students reporting harmonious family relationship showed significantly higher levels of self-esteem. This result is consistent with the findings of previous studies such as the study by Brown et al. (45). If the family relationship is unstable, the family environment is full of conflicts, it easily leads to insecurity and the children may exhibit lower self-esteem (46). Moreover, the result of this study suggested that the students with a higher level of family economic status scored higher on self-esteem. In some studies, it was referred to that the level of individual self-esteem is somehow related to their social-economic status [e.g., Ref. (47, 48)]. The higher the social-economic status and the more favorable the living conditions are, the more social resources one can get, thus a person might exhibit a higher self-esteem.

The correlation analysis showed that such family variables as only child, family relationship, grandparenting, and family income lightly correlated with level of self-esteem, while higher dysfunctional levels on all of the dimensions of the FAD were moderately related to poorer self-esteem, which was consistent with the previous studies by Krug et al. (28), Guo (49), and Guo (30). Additionally, the impact of four dimensions of the FAD (roles, communication, behavioral control, and problem solving) on self-esteem of college freshmen could be revealed in the multivariate regression analysis.

The roles dimension reflects the effect of assignment and completion of the household tasks. The household tasks include the allocation of resources (food, clothing, living, and transportation), raising and support, training of life skills, maintenance, and management of the family system (31). The findings of this study indicate that a good function of family roles has a positive impact on college students’ self-esteem. This result is consistent with the research by parts of the research by Guo (49) that examined the interactive relationship within the family function, self-esteem and interpersonal trust of junior high school students. Studies conducted by Zhou (50) and An et al. (51) also emphasize the positive relationship between clear, appropriate family roles, and high level of self-esteem. The findings of the present study confirmed the previous studies findings that while adolescents entering the adult world require independency and autonomy, they also need psychological support and approval from their parents. Thus, the family should according to the developmental stage of the offspring constantly adjust the roles and tasks of the family members to help the offspring establish high self-esteem. Another finding of this study was the relationship between communication dimension and self-esteem. The higher dysfunctional of communication is observed to promote poorer self-esteem. This was in line with the results of Zhou (50) and An et al. (51). Communication refers to the content and effectiveness of the information exchange between family members (31). Communication is an important part of family life. Family members express feelings and needs through verbal and non-verbal communication as well as transmission of information, and even build up self-awareness and self-evaluation through communication. Therefore, within a family’s communication, the clear verbal content, as well as the direct and frank way of expression, will play a positive role in promoting the level of self-esteem. In addition, the findings of this study showed that both of behavioral control dimension and problem solving dimension are predictors for the level of self-esteem. The results suggest that the functional behavioral pattern of a family in different situations for the management of individual behavior, as well as the ability of a family to respond to emergency situations and to solve problems, can have positive effects on the offspring’s self-esteem. These results are consistent with the results of relevant studies (49–52).

One of the features of this study, compared to other studies, is that the significant prediction for self-esteem of college freshmen was found among four dimensions of family functioning, namely, roles, communication, behavioral control, and problem solving. However, no other study was found in this regard. Therefore, specific dimensions of family functioning may be a valuable target for enhancing college students’ self-esteem and improvement of mental health.

There are some limitations in this study. The first limitation lies in the cross-sectional design which could not draw causality relations. Future researchers could use a longitudinal or experimental design in order to provide a better understanding about the relationship between these variables from a developmental perspective. The second limitation is that the data in this study were collected only using self-report measures, which could cause threat to the internal validity. The chosen method bears the potential risk of self-report biases, such as social desirability. For future research, we suggest to use multiple methods for evaluation (e.g., parent or peer reports) in order to minimize the influence of subjectivity. The third limitation is that the quantitative research methods could not fully reveal the impact of the unique family variables in Chinese culture, so the qualitative study design is needed in this regard in further research.

Notwithstanding these limitations, the present study provides insight into understanding the impacts of family factors on the self-esteem among Chinese college freshmen. Such specific dimensions of family functioning as family roles, communication, behavioral control, and problem solving are found to affect the self-esteem of the college freshmen. These findings suggest that the program focusing on improving family functioning at university is needed, in order to enhance the self-esteem of the college students and hence contribute to promoting the mental health and adaptation at university.

## Ethics Statement

Our study had ethics approval from the School of Medicine Ethics Committee, Tongji University, Shanghai, China. Reference number: 2014YXY16 and we acquired written informed consent from participants of the study.

## Author Contributions

JS contributed to study design, recruitment of participants, data analysis, and interpretation and writing of the manuscript. LW contributed to recruitment of participants and interpretation of results. YY contributed to recruitment of participants. FC contributed to study design and interpretation of results. NS contributed to recruitment of participants. XZ contributed to study design and interpretation of results. All authors have approved the final manuscript.

## Conflict of Interest Statement

The authors declare that the research was conducted in the absence of any commercial or financial relationships that could be construed as a potential conflict of interest.
